# Cerebrospinal Fluid Diversion for Refractory Intracranial Hypertension: A United Kingdom and Ireland Survey on Practice Variation

**DOI:** 10.7759/cureus.25877

**Published:** 2022-06-12

**Authors:** Yasir A Chowdhury, Andrew R Stevens, Wai C Soon, Emma Toman, Tonny Veenith, Ramesh Chelvarajah, Antonio Belli, David Davies

**Affiliations:** 1 Neurosurgery, Queen Elizabeth Hospital Birmingham, Birmingham, GBR; 2 Neurosurgery, Royal Stoke University Hospital, Stoke-on-Trent, GBR; 3 Critical Care, Queen Elizabeth Hospital Birmingham, Birmingham, GBR

**Keywords:** traumatic brain injury, intracranial hypertension, lumbar drainage, csf drainage, csf diversion, raised intracranial pressure

## Abstract

Introduction

Diversion of cerebrospinal fluid (CSF) in a traumatic brain injury (TBI) is an established means for achieving control of intracranial pressure (ICP), aimed at improving intracranial homeostasis. The literature and anecdotal reports suggest a variation in practice between neurosurgical centres internationally, with current guidelines advocating ventricular drainage over lumbar drainage. We sought to establish the current neurosurgical practice in the United Kingdom regarding the methods of ICP control in TBI.

Methods

A 20-point survey was distributed electronically to British and Irish neurosurgeons after ratification by the Society of British Neurological Surgeons. Questions were directed at the clinician’s opinion and experience of lumbar drain usage in patients with TBI: frequency, rationale, and experience of complications. Questions on lumbar drain usage in neurovascular patients were asked for practice comparison.

Results

Thirty-six responses from 21 neurosurgical centres were returned. Twenty-three per cent (23%) of responders reported using lumbar drains for refractory ICP in TBI patients: six units use lumbar drains and 15 do not. Three units showed partial usage, with mixed “yes/no” responses between consultants. Concerns of tonsillar herniation and familiarity with EVD were commonly given reasons against the usage of lumbar drains. Fifty-six per cent (56%) reported use in neurovascular patients.

Conclusion

This contemporary practice survey demonstrates mixed practice across the UK and within some centres. Responses and survey feedback demonstrate that the use of lumbar drains in TBI is a polarising topic. The variety of practice between and within neurosurgical units supports consideration of the prospective study of CSF diversion methods for control of refractory ICP in patients with TBI.

## Introduction

Background

Moderate and severe traumatic brain injury (TBI) management is centred on minimising secondary brain injury. In the critical care setting, this is principally based on avoiding raised intracranial pressure (ICP) whilst maintaining adequate cerebral perfusion pressure (CPP) [1].

The Brain Trauma Foundation (BTF) guidelines provide a strategy for sequential escalation of therapeutic intensity until ICP is controlled [1]. Initial medical treatments include sedation, mild hypocapnia and hyperosmolar therapy. Once these interventions fail to control intracranial hypertension, advanced interventions, including cerebral spinal fluid (CSF) diversion, barbiturate coma and decompressive craniectomy can be considered.

CSF diversion

CSF diversion to control ICP is based on the Monro-Kellie doctrine [2-3]. In the context of TBI, diversion (or buffering) of CSF allows the increase of parenchymal oedema from trauma within physiological ICP. CSF diversion occurs via two access points: (1) a ventriculostomy in the lateral ventricle connected to an external ventricular drain (EVD) and (2) a lumbar catheter connected to an external lumbar drain (ELD).

Anecdotally, there is significant variation in practice over the use of EVD or ELD to manage refractory ICP in TBI. CSF diversion in TBI is primarily achieved through a ventriculostomy. Although an EVD also permits the measurement of intraventricular pressure, ICP is now more commonly measured via a transcranial intraparenchymal ‘bolt’ [4]. In paediatrics centres, ICP monitoring via an EVD is more commonly used over an ICP bolt in TBI [5]. ELD was developed in the 1960s to reduce cerebral tension intraoperatively [6] and is now an established method of CSF diversion in a variety of settings [7], including post-traumatic CSF leak [8-9], normal pressure hydrocephalus assessment [10], skull base surgery [11-12], and in thoracoabdominal aortic surgery to reduce spinal cord ischemia [13].

Drainage of CSF via a lumbar puncture or lumbar catheter is associated with a risk of transtentorial herniation where there is impeded CSF flow across the foramen magnum [14-17]. Furthermore, EVD has been recommended by a recent consensus statement whilst ELD has not gained this support. Despite this, there are anecdotal reports of ELD usage for ICP control in TBI in a number of United Kingdom (UK) neurosurgical centres. More established in the literature, ELD has been adopted for use for intracranial hypertension in the setting of bacterial and cryptococcal meningitis [18-19] and subarachnoid haemorrhage (SAH) [20-22].

Current evidence

Our systematic review of the use of ELD in ICP control in TBI found 159 patients across nine studies [23]. The review identified three studies, which combined TBI and vascular patients, finding that, in total, 159 patients with TBI had been reported in the literature. Efficacy in gaining ICP control with ELD was well-established, with a possible effect when used in addition to EVD. The quality of safety reporting varied across the studies, with 14 cases reported of clinical or radiological cerebral herniation in 230 patients. Reviewers identified one included patient in whom ELD had been reported to have resulted in patient morbidity, though not an adverse outcome. Delineation of the effect of lumbar drain from the progression of disease in this cohort is inherently difficult to establish, but included study authors had not associated ELD usage with these outcomes. The review concluded that whilst there is no evidence to suggest an adverse safety profile, the data in the literature was deemed insufficient to conclusively confirm safety. The data did not allow quantifying the rate of tonsillar herniation associated with ELD. Badhiwala et al., in their meta-analysis, reviewed 110 patients across six studies undergoing ELD for traumatic intracranial hypertension [24]. They also found ELD aids in reducing ICP but there is a paucity of evidence on the duration of effect and safety across its use in all patients. These systemic reviews in combination conclude that there is a cohort of patients with refractory traumatic intracranial hypertension (a minimum of 20 mmHg) with open basal cisterns and no intracranial lesion where ELD may be used safely.

Aims

There are anecdotal reports of varying UK practices of CSF diversion in TBI, thus we sought to establish the variation in practice amongst United Kingdom (UK) and Ireland neurosurgical centres, and explore the experiences of neurosurgeons on the usage of ELD for ICP control in TBI. Our primary objective was to establish the variation in practice in the use of CSF diversion in TBI amongst UK neurosurgery centres. Our secondary objectives were to qualitatively assess: (1) the practitioner experience of ELD; and (2) the rationales employed in informing their individual and local practice, using both TBI and neurovascular pathologies as context for discussion.

## Materials and methods

A 20-point survey (see Appendix 1) was constructed to identify the practice, rationale and experience of UK neurosurgeons on the use of ELD in patients with TBI. Questions were based on: the use of ELD in TBI; frequency; indication/exclusion; rationale; standard operating procedure (SOP); and any encountered complications. Free space was provided for comment, and respondents were asked to indicate their interest in participation in prospective observational studies. To explore more widely the experiences and practice variation in the use of ELD, respondents were asked about the relevance of the use of ELD in neurovascular patients. Whilst the pathophysiological process and directed management differ greatly from TBI, the practice could carry the possibility of similar adverse events, and we sought to explore practitioner opinions in both contexts.

The draft survey was submitted to the Society of British Neurological Surgeons (SBNS) Academic Committee, which performed the appropriate national institutional board review. The committee advised productive revisions and approved the national survey. The questionnaire was created using a Google Form and a link to the survey was emailed to members of the SBNS. This included members working in 40 neurosurgical units in the UK and Ireland. Responses were uploaded directly online and were subsequently collated. A qualitative analysis of the results was performed. As this was a voluntary survey of healthcare professionals, no ethical approval was required.

## Results

Responses

**Figure 1:**
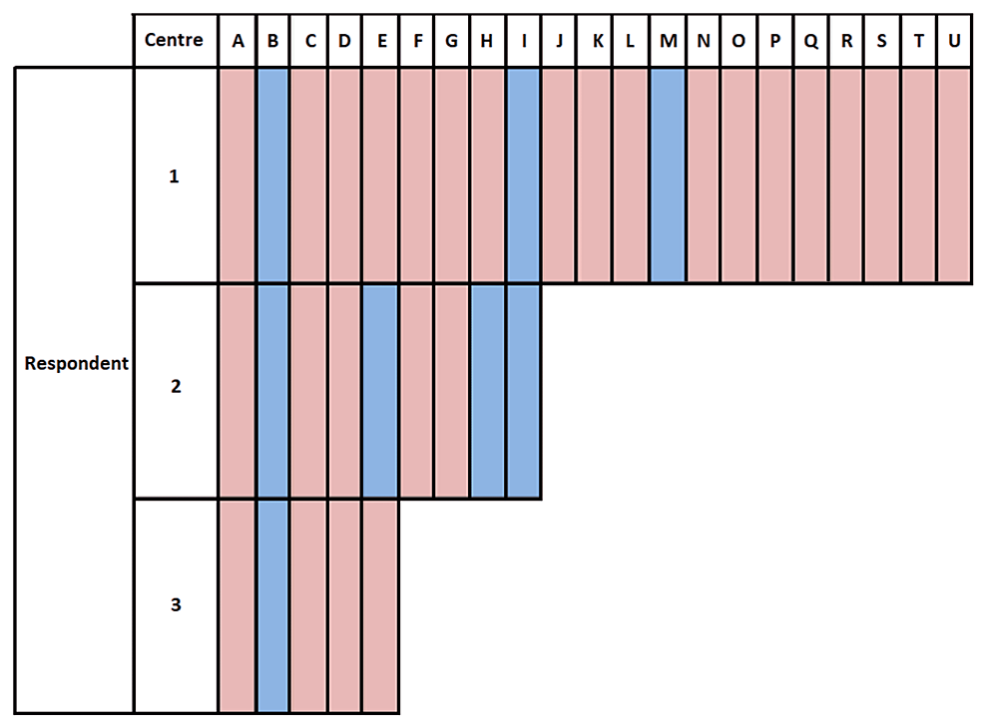
Summary of respondent responses to “Does your unit use lumbar drains for the treatment of refractory ICP in TBI patients?” Centres are anonymised by codes A-U, and respondents coded 1-3. Blue = yes; Red = no.

Thirty-six questionnaires were completed, which included 30 consultants and six neurosurgery trainees. Responses came from 21 of 40 neurosurgery units across the United Kingdom and Ireland. Two paediatric units responded, and one non-major trauma centre unit responded. Trainees completing the survey confirmed they had discussed the returned form with a consultant within the department when answering questions. Nine responders from six units reported using ELD in TBI patients for ICP control: all responders from three units and a mixed (yes/no) response from three units. Overall, 23% of responders reported using lumbar drains for refractory ICP in TBI patients (Figure 1). One unit reported a frequency of one to two per month. All other responses of frequency were “less than once per month”. No unit reported having a standard protocol for the practice.

Indications and exclusions

Units reporting to use ELD in TBI gave a variety of indications and considerations. Indications were quoted as: “severe TBI with diffuse oedema”; “ICP 20-30”; “ICP 25-30”; “ongoing ICP issues or cerebral herniation through a craniectomy site”; “high ICP”; “refractory ICP”; and “continuing hydrocephalus after two lumbar punctures”.

Exclusion criteria quoted were: “no post-fossa haematoma and no midline shift”; “no large mass lesions especially infratentorial”; “not tight at tentorial insura or foramen magnum”; “risk of herniation”; “no evidence of brain herniation, basal cisterns open and no dubiety about space at foramen magnum, also would not use if unevacuated mass lesion”; “no supratentorial mass lesion”; “no mass lesion”; and “patent basal cisterns and cisterna magna”.

Rationale for not using

A variety of responses were given as reasons for not using ELD for either TBI or vascular patients. Some cited the use of EVD: “thought [ELD] ineffective versus EVD”; “we tend to use EVD instead”; “EVD first line in emergency”; “safer… with EVD”; “EVD more common practice”; “EVD is safer”; “EVDs are easy to insert”. “high infection” “to avoid syrinx”; and “coning”, “herniation”, ”craniospinal gradient” (or variations thereof, n = 7).

Timing of CSF diversion

**Figure 2:**
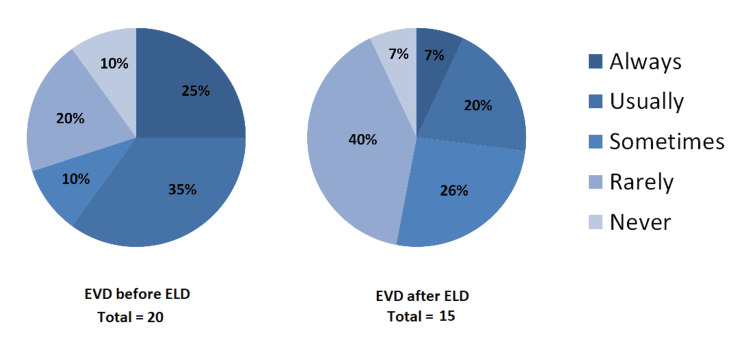
Pie charts showing when ELD is considered in relation to EVD “Not applicable” responses are not shown (20 for EVD before ELD (left pie chart), 15 for EVD after ELD (right pie chart)). EVD: external ventricular drain; ELD: external lumbar drain

Respondents were asked questions on the order of precedence for lumbar drainage and EVD prior to compressive surgery. Of respondents that reported the use of ELD in their unit, when asked if an EVD would be inserted prior to an ELD, n = 7 reported “usually”; n = 2 reported “sometimes”; n = 4 reported “rarely”. With a lumbar drain in place, prior to considering surgical decompression, of those respondents that reported the use of ELD, when asked if they would consider using an EVD as an alternative or additional means of CSF diversion, n = 3 reported “usually”; n = 4 reported “sometimes” and n = 6 reported “rarely”. Figure 2 shows further results for this question.

Comparison with vascular patients

As described above, 23% of responders reported using lumbar drains for refractory ICP in TBI patients. In vascular patients, 56% of responders reported using lumbar drains. Frequency was also greater: 52% reported usage of once a month or more in vascular patients compared with 33% in TBI patients. When asked for rationale when using ELD for vascular patients and not TBI patients, responses were varied. Respondents reported: “more familiarity with EVD”; “different pathology”; “will use only for CSF flow”; “would use in communicating hydrocephalus” (or variations thereof, n = 5); and “concerns of tonsillar descent/coning” (or variations thereof, n = 5).

Responses were similar when asked for indications for using ELD in vascular patients: “(communicating) hydrocephalus” (or variations thereof, n = 15); “raised ICP”; “to improve perfusion pressure”; “vasospasm” or “neurological deficit” (n = 3); “stepdown from EVD”; and “headache in awake patients”.

For exclusion criteria, respondents discussed: “obstructive hydrocephalus” “fourth ventricular blood” or “effaced basal cisterns” (n = 13); “unsecured ruptured aneurysm”; “bleeding diathesis”; “familiarity”; and “patient cooperation”.

Reported experience of complications

**Figure 3:**
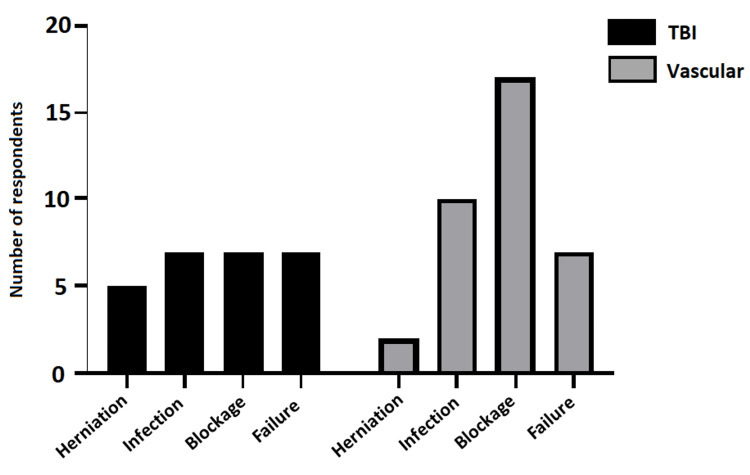
Bar graph showing response to “…have you encountered any of the following complications in your unit?” for ELD use in TBI and vascular patients Frequency of multiple-choice (‘tick all that apply’) responses shown (n = 19 responses, not including “Not applicable” (n = 13)). ELD: external lumbar drain; TBI: traumatic brain injury

Five of 19 respondents reported having encountered tonsillar herniation after ELD usage in TBI and two of 19 respondents in vascular settings. Reported blockage incidence was reported as higher in vascular (n = 17) patients than TBI (n = 7). Figure 3 shows the range of reported complications.

## Discussion

This qualitative national practice survey has demonstrated considerable practice variation in the use of lumbar drainage in ICP control in TBI, with mixed opinion and practice across the UK and Ireland. There is also evidence of practice variation within neurosurgical centres. Current practice is demonstrated to predominantly utilise EVD rather than lumbar drainage for ICP control in TBI. Overall, 23% of responders reported using lumbar drains for CSF diversion to treat medically refractory ICP in TBI patients. Based on responses of annual unit frequency, we estimate that 15-30 lumbar drains are inserted per year nationally for ICP control in TBI, yet there is a clear prevalence of safety concerns with their use.

Safety concerns about lumbar drainage were the overriding aspect that respondents quoted when discussing their choice of CSF diversion method. Practitioners using EVD discussed the possibility of lumbar drainage creating a craniospinal gradient, particularly where there is effacement of the basal cisterns or impeded flow of CSF across the foramen magnum, which was a specific concern raised. Similarly, those respondents who use lumbar drainage for TBI cited such circumstances would preclude its use in their practice. Therefore, the predominant main concerns from non-users are shared by users. This highlights that there are clear circumstances, agreed across respondents, where lumbar drainage is not safe, in keeping with the selection criteria of studies utilising this method in the literature [23-24]. This also highlights prevalent concerns about the safety profile of ELD amongst practitioners.

Experienced or perceived complications are not reliable measures of incidence, and this questionnaire has not been designed to draw valid conclusions on the safety of CSF diversion methods. This methodology however provides some insight into the experiences and perceptions of clinicians. Whilst statistical analysis was not appropriate with the present methodology, 14% of respondents reported previous clinical experience in their neurosurgical unit of transtentorial herniation after the use of ELD in patients with TBI compared with 6% in neurovascular patients. Without further detail on the context of these cases, this alone does not offer usable evidence in informing safety of ELD but does contextualise the practice variation. As a further contrast of experience between ELD and EVD, a relatively higher experienced incidence of blockage in vascular patients was reported; this stands to reason given the narrow lumen of ELD compared with EVD and the preponderance of blood-stained CSF in the vascular patient compared with TBI.

No survey response reporting the use of ELD in TBI patients stated that their department had an established protocol. The common rationale for using ELD was in patients who had intracranial hypertension but no surgical haematoma in the posterior fossa with no radiological evidence of tonsillar herniation. As identified by systematic reviews in the literature, protocols varied greatly between studies [23-24]. The lack of standard operating procedures or locally accepted policy is unlikely to support clinicians to utilise ELD in circumstances that they may otherwise deem preferable to EVD. Similarly, this perpetuates a lack of familiarity with their use and requires the clinician to determine the drainage protocol (pressure/volume-guided drainage and their respective values).

Limitations

This study is limited by a number of factors. The response rate was small but comparable with that expected in the literature based on recent studies with a similar methodology [25-26]. Despite this, there was good regional representation and responses from over half of all neurosurgical units in the UK. Assuming units who did not respond to the survey do not use ELD for TBI, at least 15% (6 out of 40) of units across the UK and Ireland have clinicians using ELD for TBI. Extrapolation to the international context is limited.

A further limitation is that the premise of this survey is based on the anecdotal observations of ELD use in TBI patients, as this represents a variation from guidance. As such, the questions are principally directed at understanding the experience, use and practice of ELD; the use of EVD in this context may only be inferred. Comparison with vascular patients was included due to similar anecdotal variation in practice though with more utilisation of lumbar drainage in this cohort than in TBI. This inclusion, however, created some doubt amongst a few respondents as to the focus of the survey. Further, these questions (to retain comparison with TBI patients) were directed at general “intracranial hypertension” in vascular patients. The open-ended questions led to corresponding responses on the pathophysiology of communicating hydrocephalus in the vascular patient.

Overall attitudes to ELD use were mixed. The questionnaire asked respondents whether they would be interested in participating in a prospective study on the use of ELD in refractive ICP in TBI. Answers were mixed: Yes = 10; No = 6; Maybe = 10. Open opportunity for comment was given and responses varied significantly, demonstrating polarised views within the UK and Ireland neurosurgical community. One respondent was “not at all keen on this as an idea” whilst another “would support a move towards more lumbar drainage”, as examples of the elicited spectra of opinion. A similar polarity of opinions was noted amongst general free-text responses. Two respondents cited the need for more evidence.

Future research

This national practice survey has identified considerable practice variation and polarised views on the methods of CSF diversion in TBI. As identified in the literature, there is insufficient evidence to determine the safety of ELD use for ICP control in patients with TBI. However, this study has identified that ELD is used with modest prevalence in contemporary UK neurosurgical practice. As such, this supports the need for further evidence to compare CSF diversion methods to understand the safety, efficacy and relative merits of EVD and ELD. In designing a prospective study methodology, differentiating disease progression of TBI from iatrogenic complications is challenging where the clinical outcomes (tonsillar or cerebral herniation) are the same. Any potential prospective study on the use of lumbar drains should have clear and robust adverse event reporting procedures to ensure that any “true” complication is not missed for this reason. This survey has identified that users of ELD have strict inclusion criteria for suitability in accordance with the literature: any future study should reflect such strict criteria to ensure both patient safety and the scientific value of results.

## Conclusions

We have demonstrated the significant variation in practice across the UK and within neurosurgical units when considering the methods of CSF diversion for refractory ICP control in TBI. Principle justification quoted by non-users of ELD is based on the possibility of transtentorial herniation, and practitioners regularly using ELD also reference the risk of cerebral herniation in their exclusion criteria. Further evidence is required to determine whether ELD is a safe method of CSF diversion for patients with TBI, but its prevalence in modern practice identifies the need for further evidence to validate or reject its ongoing use. Given the significant regional practice variation, there appears to be suitable frequency and sufficient support for prospective study to compare ELD versus EVD.

## Appendices

Appendix 1

1.    Which unit do you work in?

☐ Aberdeen

☐ Alder Hey Children's (Liverpool)

☐ Barking, Havering & Redbridge Hospitals

☐ Beaumont Hospital (Dublin)

☐ Birmingham Children's Hospital

☐ Brighton & Sussex

☐ Bristol

☐ Cambridge

☐ Cardiff

☐ Children's University Hospital (Dublin)

☐ Cork

☐ Coventry

☐ Dundee

☐ Glasgow

☐ Great Ormond Street Hospital (London)

☐ Hull

☐ Imperial College (London)

☐ King's College London

☐ Leeds

☐ Middlesbrough

☐ Newcastle

☐ Nottingham

☐ Oxford

☐ Plymouth

☐ Preston

☐ Queen Elizabeth Hospital Birmingham

☐ Queen Square (London)

☐ Royal Hallamshire (Sheffield)

☐ Royal Hospital for Sick Children (Edinburgh)

☐ Royal London Hospital

☐ Salford Royal (Manchester)

☐ Sheffield Children's Hospital

☐ Southampton

☐ Stoke

☐ St.George's Hospital (London)

☐ Walton (Liverpool)

☐ Western General (Edinburgh)

2.    Please confirm your role within the unit

☐Consultant Neurosurgeon

☐Neurosurgery Clinical Fellow

☐Neurosurgery Trainee

3.    If you are a fellow or trainee please confirm you have consulted with a Consultant Neurosurgeon within your unit:

☐ Yes

☐ N/A

4.    Does your unit use lumbar drains for the treatment of refractory ICP in TBI patients?

☐ Yes

☐ No

5.    Does your unit use lumbar drains for the treatment of refractory ICP in vascular patients?

☐ Yes

☐ No

6.    If you do use lumbar drains for ICP control in TBI patients, what is the estimated frequency of lumbar drain use?

☐ Less than once a month

☐ Once a month

☐ Twice a month

☐ More than twice a month

☐ N/A

7.    If you do use lumbar drains for ICP control in vascular patients, what is the estimated frequency of lumbar drain use?

☐ Less than once a month

☐ Once a month

☐ Twice a month

☐ More than twice a month

☐ N/A

8.    If you don't use lumbar drains for ICP control in TBI or vascular patients, what are the reasons for not using them?

(free text box)

9.    If you use lumbar drains for ICP control only in vascular patients, what are the reasons for not using them in TBI patients?

(free text box)

10. What would you consider your indications, inclusion and exclusion criteria for lumbar drain insertion in vascular patients?

(free text box)

11. What would you consider your indications, inclusion and exclusion criteria for lumbar drain insertion in TBI patients?

(free text box)

12. Does your unit have a lumbar drainage protocol in the setting of ICP control in TBI or vascular patients?

☐ Yes

☐ No

☐ N/A

13. If yes to above, what is the protocol?

(free text box)

14. If lumbar CSF drainage is used for management of refractory ICP, have you encountered any of the following complications in your unit? Tick all that apply:

TBI Patients:

☐ Tonsillar herniation

☐ Infection

☐ Blockage needing revision / re-insertion

☐ Failure of ICP control needing further intervention

☐ N/A

Vascular patients:

☐ Tonsillar herniation

☐ Infection

☐ Blockage needing revision / re-insertion

☐ Failure of ICP control needing further intervention

☐ N/A

15. In refractory ICP in vascular patients, prior to lumbar drain insertion would an EVD be inserted?

☐ Always

☐ Usually

☐ Sometimes

☐ Rarely

☐ Never

☐ N/A

16. In refractory ICP in TBI patients, prior to lumbar drain insertion would an EVD be inserted?

☐ Always

☐ Usually

☐ Sometimes

☐ Rarely

☐ Never

☐ N/A

17. In refractory ICP in vascular patients with a lumbar drain already in place, prior to considering surgical decompression would an EVD be inserted?

☐ Always

☐ Usually

☐ Sometimes

☐ Rarely

☐ Never

☐ N/A

18. In refractory ICP in TBI patients with a lumbar drain already in place, prior to considering surgical decompression would an EVD be inserted?

☐ Always

☐ Usually

☐ Sometimes

☐ Rarely

☐ Never

☐ N/A

19.    Do you have any other comments to make about the use lumbar drain in refractory ICP in vascular and/or TBI patients?

(free text box)

20.    If lumbar CSF drainage is used for management of refractory ICP in your unit, would you be interested in participating in a multi-centre prospective observational study on the use of lumbar CSF drainage for management of refractory ICP in TBI or vascular patients?

☐ Yes

☐ No

☐ Maybe

☐ N/A
